# ﻿*Megabranchiella* gen. nov., a new mayfly genus (Ephemeroptera, Baetidae) from Thailand with description of two new species

**DOI:** 10.3897/zookeys.1125.90802

**Published:** 2022-10-18

**Authors:** Sirikamon Phlai-ngam, Boonsatien Boonsoong, Jean-Luc Gattolliat, Nisarat Tungpairojwong

**Affiliations:** 1 Department of Biology, Faculty of Science, Khon Kaen University, Khon Kaen 40002, Thailand; 2 Animal Systematics and Ecology Speciality Research Unit (ASESRU), Department of Zoology, Faculty of Science, Kasetsart University, Bangkok 10900, Thailand; 3 Museum of Zoology, Palais de Rumine, Place Riponne 6, CH-1005 Lausanne, Switzerland; 4 Department of Ecology and Evolution, University of Lausanne, CH-1015 Lausanne, Switzerland; 5 Applied Taxonomic Research Center (ATRC), Faculty of Science, Khon Kaen University, Khon Kaen 40002, Thailand

**Keywords:** Baetidae mayflies, enlarged gills, South East Asia, taxonomy

## Abstract

*Megabranchiella***gen. nov.** (Ephemeroptera: Baetidae) is established as a new baetid mayfly genus from northern Thailand. Two new species, *Megabranchiellascutulata***sp. nov.** and *Megabranchiellalongusa***sp. nov.**, are described. This genus is distinguished from other Baetidae by abdominal segment I, bearing a pair of enlarged, ventrally oriented single gills, covering abdominal sternites II–V; other gills have normal size and are dorsolaterally oriented. The two new species *Megabranchiellalongusa***sp. nov.** and *Megabranchiellascutulata***sp. nov.** can be differentiated by the setation of femur dorsal margin and the shape of abdominal gill I. This mayfly genus was found in flowing water with cobble microhabitats in headwater streams of northern Thailand.

## ﻿Introduction

Baetidae is a common family of mayflies with a worldwide distribution, except for Antarctica and New Zealand. The family comprises ca. 1,070 species assigned to 110 genera; it comprises approximately one-third of the global mayfly diversity and is therefore the most diversified mayfly family (Sartori and Brittain 2015; Jacobus et al. 2019; Kaltenbach et al. 2020). The generic diversity of Baetidae is the highest in the Afrotropical realm (ca. 40 genera), followed by the Oriental and Neotropical realms (ca. 28 and 27 genera, respectively), and finally the Nearctic and Palearctic realms (ca. 20 genera and 17 genera, respectively), while the lowest diversity is in the Australasian realm (ca. 12 genera) (Gattolliat and Nieto 2009; Kaltenbach et al. 2020). Although the Oriental realm has several regions with high potential diversity (e.g. the Indian subcontinent, Sunda islands and the Philippines), the apparent low diversity most certainly reveals a lack of data and poor sampling (Kaltenbach et al. 2021). The knowledge of the diversity in the Oriental realm, including Southeast Asia has been gradually improved in the last two decades. New genera and new species have been described and reported from many islands, including Indonesia, New Guinea, Philippines, and Malaysia (Sabah) (Kaltenbach et al. 2020, 2021). Despite these recent improvements, the real extent of the diversity remains only partially known and the distribution of most species underestimated (Suttinun et al. 2020).

Three new genera have been described from Southeast Asia in recent times. A new genus, *Procerobaetis* Kaltenbach & Gattolliat, and four new species, *P.leptobranchius* Kaltenbach & Gattolliat, 2020 *P.petersorum* Kaltenbach & Gattolliat, 2020 *P.fretagi* Kaltenbach & Gattolliat, 2020 and *P.totuspinosus* Suttinun, Kaltenbach & Boonsoong were, 2021 reported from Indonesia (Sumatra), Philippines and Thailand (Kaltenbach et al. 2020; Suttinun et al. 2021). *Philibaetis* Kaltenbach & Gattolliat, a new baetid mayfly genus, was also reported from the Philippines with two species, *P.luzonensis* (Müller-Liebenau, 1982) and *P.realonae* (Müller-Liebenau 1982) (Kaltenbach et al. 2021). Since the last decade, the trend in species diversity of the Baetidae has also been increasing in Thailand, due to recording of new taxa or distribution extension. Approximately nine genera and 13 species have been reported and described from Thailand (Suttinun et al. 2021). The newest genus, *Cymbalcloeon* Suttinun, Gattolliat & Boonsoong, with a new species, *Cymbalcloeonsartorii* Suttinun, Gattolliat & Boonsoong, 2020 was reported as a Thai endemic (Suttinun et al. 2020). The present work describes a new genus of Baetidae from Thailand. This new genus was collected during a large survey of Baetidae from Northern Thailand. It was collected in headwater streams. Herein, the new genus and two new species were described at the larval stage. The comparison with the larval morphology of other genera is also provided.

## ﻿Materials and methods

Larval specimens were collected by hand-picking from all stream orders in Northern Thailand. Sampling sites are located in various natural conditions. *Megabranchiella* larval specimens were found and collected from headwater streams in the northern region only (Table 1; Fig. 15).

**Table 1.** GPS coordinates of locations of examined specimens.

Species	Provinces	GPS coordinates	Altitudes (m a.s.l.)
*M.scutulata* sp. nov.	Chiang Mai	18°52'01.65N, 99°19'20.83E	779
	Chiang Rai	20°00'39.60N, 99°48'14.47E	476
*M.longusa* sp. nov.	Chiang Mai	18°32'50.02N, 98°30'49.79E	1,359
	Nan	19°09'19.09N, 101°10'12.96E	995

The specimens were preserved in 95% ethanol. The larvae were dissected in glycerin under a Nikon SMZ745 stereomicroscope, with subsequent mounting on slides by glycerin. The micro-characters were observed with a Nikon Eclipse E200LED MV J compound microscope. Drawings were prepared using a camera lucida attached to an Olympus CH30 compound microscope, and they were scanned for illustration with the Procreate application (iOS application). Photographs of larvae were taken with a Canon EOS 700D camera and edited with Adobe Lightroom (http://www.adobe.com). The habitus photographs and measurements (given in mm) were obtained using NIS-Elements software with a Nikon SMZ25 stereomicroscope. Final plates were prepared and processed with Adobe Photoshop (http://www.adobe.com). For scanning electron microscopy (SEM), specimens were preserved in 95% ethanol and transferred to absolute ethanol for dehydration. The specimens were then dissected and transferred to microtubes and covered with a fine mesh net (mesh size 60 µm) for drying in a Critical Point Dryer (CPD). The dried specimens were set on stubs and coated with a 20 nm layer of gold with a Cressington sputter Coater. SEM images were obtained by Field Emission Scanning Electron Microscopy (FESEM; Fei Model: Helios NanoLab G3 CX). The examined material was deposited in the Collection of Aquatic Insect of Department of Biology at Khon Kaen University in Khon Kaen, Thailand (**KKU-AIC**), the collection of the Zoological Museum at Kasetsart University in Bangkok, Thailand (**ZMKU**) and the Museum of Zoology in Lausanne, Switzerland (**MZL**).

This research has been reviewed and approved by the Institutional Animal Care and Use Committee of Khon Kaen University, based on the Ethic of Animal Experimentation of National Research Council of Thailand (Record No. IACUC-KKU-65/63) for collecting the baetid mayfly specimens.

## ﻿Results

### ﻿Taxonomy

**Order Ephemeroptera Hyatt & Arms, 1891**

#### Family Baetidae Leach, 1815

##### ﻿Genus Megabranchiella

Taxon classificationAnimaliaEphemeropteraEphemeroptera

Phlai-ngam & Tungpairojwong gen. nov.

80761833-451F-5DAD-9DB6-C2143A3E4B99

https://zoobank.org/A9F66B30-FEDB-46FA-B8E7-D6A338E10D63

###### Type species.

*Megabranchiellascutulata* sp. nov., by present designation.

###### Included species.

*Megabranchiellalongusa* sp. nov.

*Megabranchiellascutulata* sp. nov.

###### Diagnosis.

**Figure 1.**
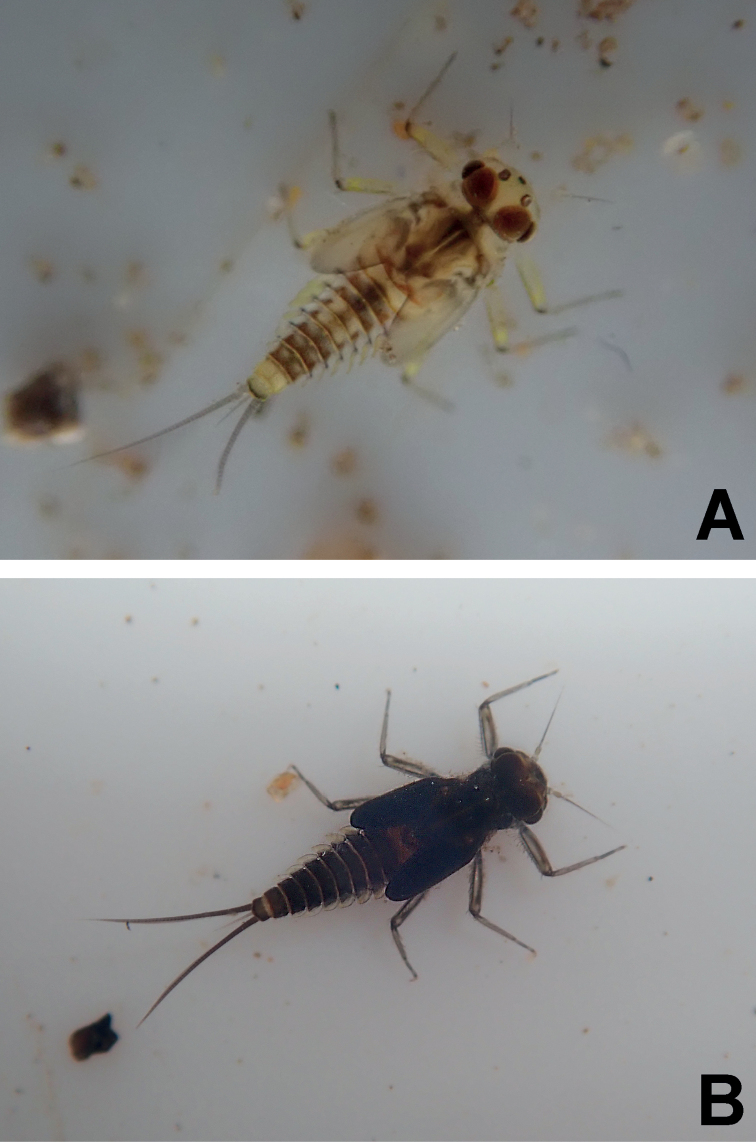
*Megabranchiellascutulata* sp. nov., male larva **A** early larval stage **B** last larval instar.

**Figure 2.**
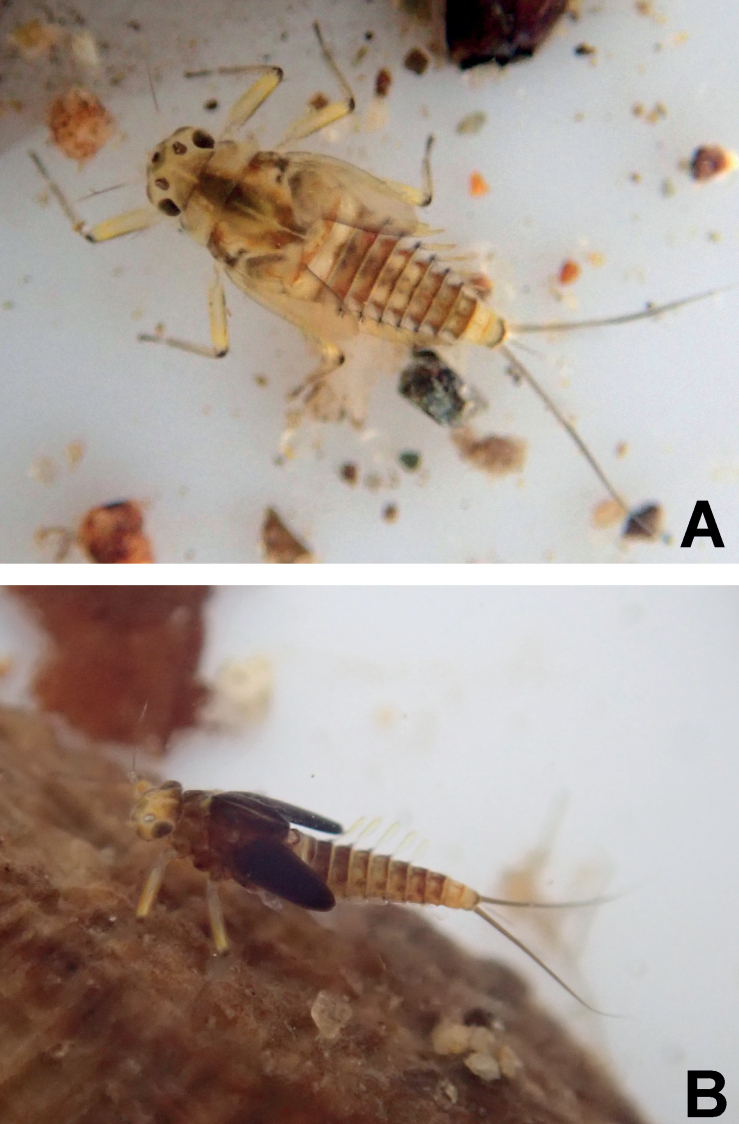
*Megabranchiellascutulata* sp. nov., female larva. **A** early larval stage **B** last larval instar.

**Figure 3.**
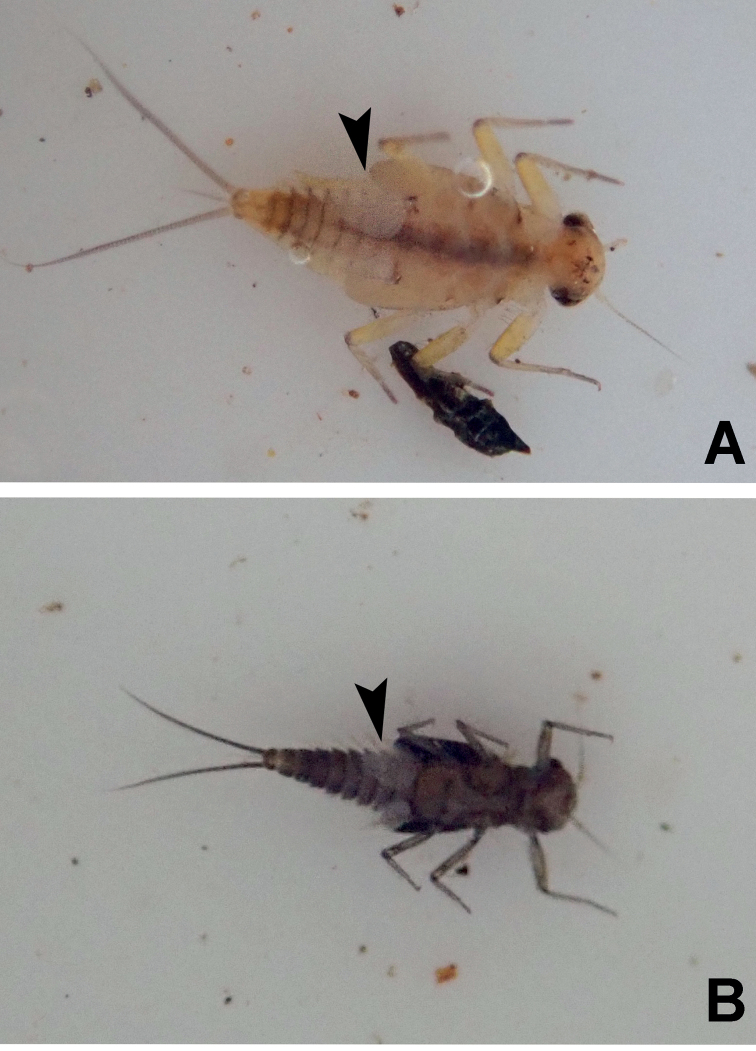
*Megabranchiellascutulata* sp. nov., ventral view **A** early larval stage **B** last larval instar. (arrow: gills I).

**Figure 4.**
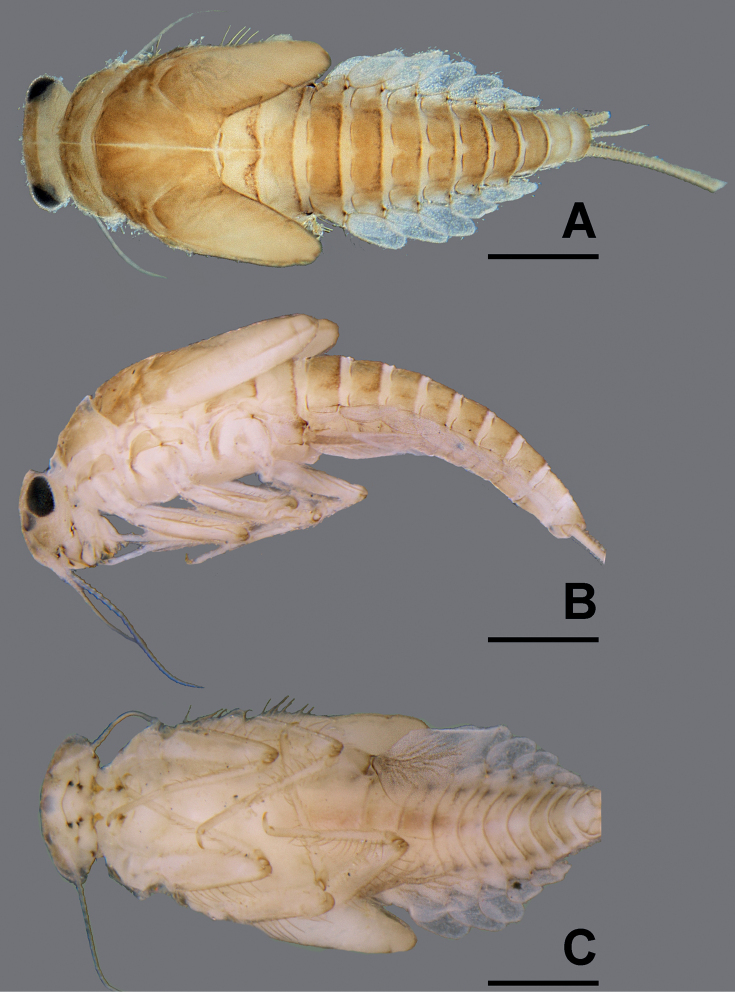
*Megabranchiellascutulata* sp. nov., larval habitus **A** dorsal view **B** lateral view **C** ventral view. Scale bars: 0.5 mm.

**Figure 5.**
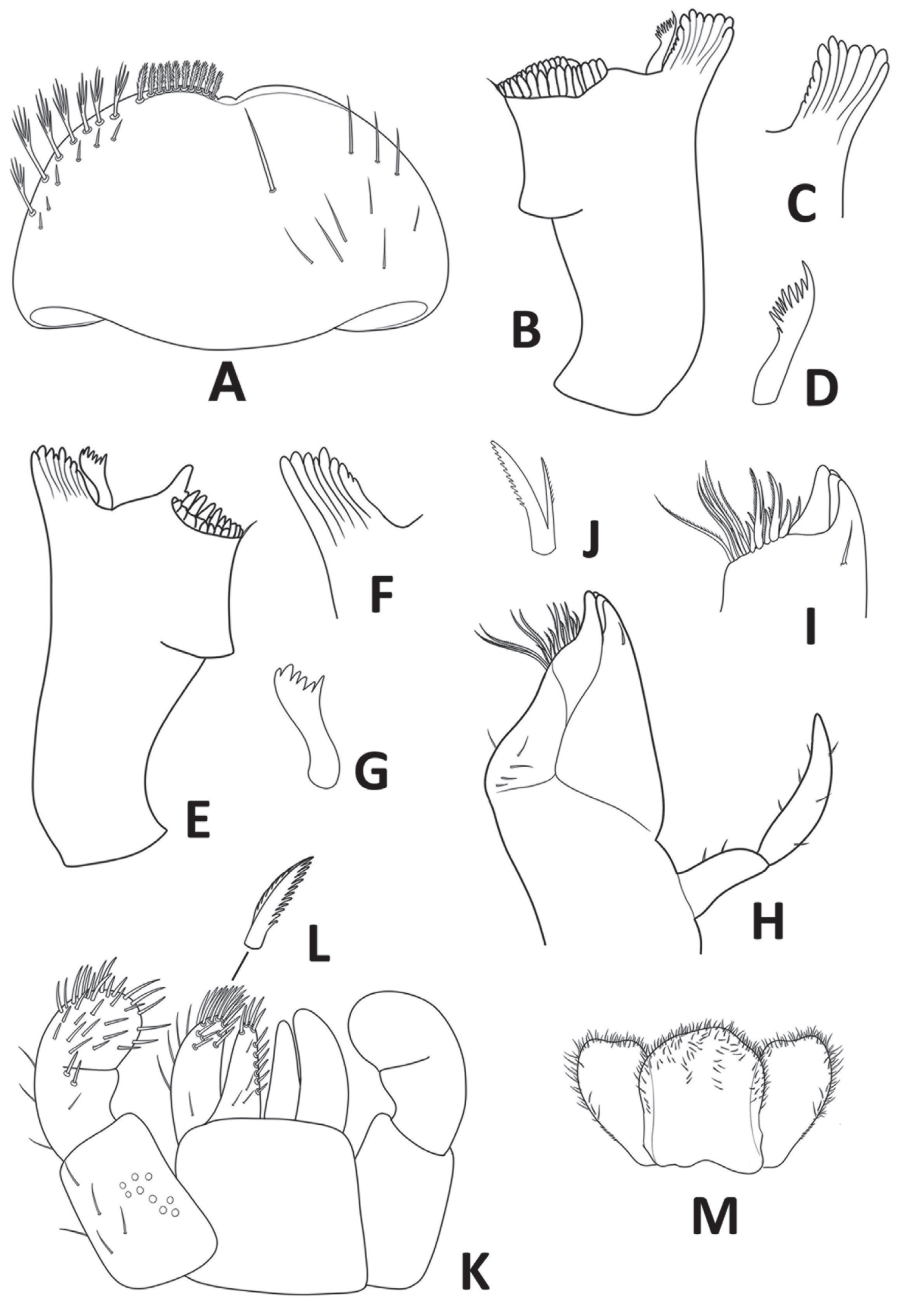
*Megabranchiellascutulata* sp. nov., larval morphology **A** labrum **B** right mandible **C** right incisor **D** right prostheca **E** left mandible **F** left incisor **G** left prostheca **H** maxilla **I** apex of galea-lacinia **J** denti-seta **K** labium **L** long, robust, pectinate setae **M** hypopharynx.

**Figure 6.**
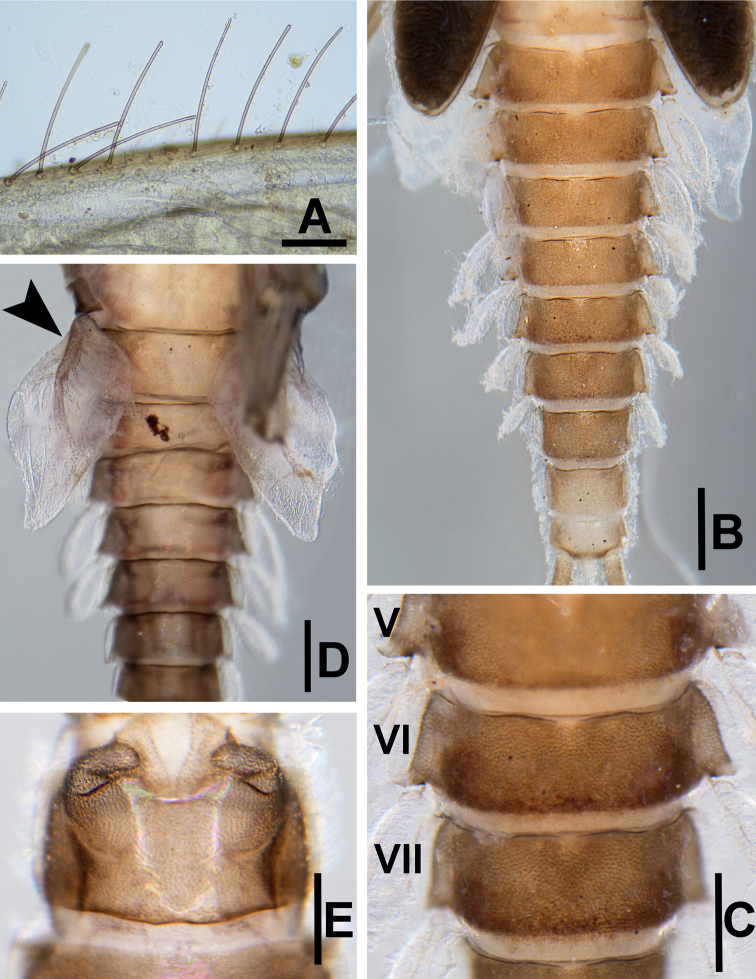
*Megabranchiellascutulata* sp. nov., larval morphology **A** dorsal margin of femur **B** dorsal view of abdomen **C** enlargement of abdominal tergites V–VII **D** ventral view of abdominal gills I (arrow) **E** ventral view of gonostyli. Scale bars: 0.15 mm (**A**); 0.5 mm (**B**); 0.25 mm (**C**); 0.4 mm (**D**); 0.3 mm (**E**).

***Larva*** (Figs 1–3). Larval body ventrally flattened (Fig. 4). Margins of head capsule relatively densely covered with fine, long setae. Antenna without process on scape, margins of scape and pedicel densely covered with long, fine, simple setae; flagellum short, covered with scattered long, fine, simple setae. Mouthparts relatively compact. Labrum (Fig. 5A) broadly rounded, dorsally with one central seta and a row of setae reduced in number. Mandibles with smooth margin between mola and prostheca, without setae, right and left prostheca comb-shaped. Maxilla (Fig. 5H) with 2-segmented palp, with a small tip at apex. Labium compact (Fig. 5K), glossa and paraglossa covered with stout setae, labial palp 3-segmented, terminal segment rounded. Thorax broad; forewing pad broad, large, divergent; hindwing pad highly reduced. Femur with a regular row of long, stout, setae on dorsal margin, surface covered with scattered tiny spine-like setae anteromedially, ventral femoral patch present. Tibia with long feathered setae on dorsal margin, covered with scattered fine setae and short, pectinate setae. Tarsus without preapical setae, tarsal claw with one row of denticles. Abdominal tergites covered with scattered long, fine setae; posterior margin smooth. Abdominal gills segment I (Fig. 6D) ventrally oriented, enlarge covering abdominal sternites II to V; gill margin smooth without setae. Abdominal gills segment II–VII dorsolaterally oriented, gill margins with densely covered with long fine setae. Gonostily under larval cuticule *Acentrella*-type (Fig. 6E). Paraproct surface with notched scales and long setae; distal margin smooth without prolongation. Caudal filaments with swimming setae; median filament reduced to shorter than 0.4× of cerci length.

###### Winged stage.

Unknown.

###### Etymology.

*Megabranchiella* is a combination of *Mega*- in reference to the enlarged, -*branchio*- in reference to gills and -*iella* in reference to the genera *Liebebiella* and *Acentrella* which are most certainly the closely related genera. The “*Megabranchiella*” refers to the remarkable enlarged abdominal gill segment I of baetid mayfly. The gender is feminine.

###### Description.

***Larva*** (Figs 1–3). ***Body*** Relatively short and ventrally flattened (Fig. 4), covered with scattered long, fine setae; head and thorax in lateral view rounded.

***Head*. *Antenna*.** ca. 2 × as long as head length; scape, pedicel and flagellum without process, without scale bases and spines, covered with scattered long, fine setae; flagellum covered with scattered long, fine setae in each segment.

***Labrum*** (Fig. 5A). Broadly rounded; wider than long; dorsal surface with one central seta and a row of setae reduced in number, long scattered simple setae along dorsal margin, scattered simple, hairlike setae; distal margin with anteromedian notch shallow, disterolateral margin with long feathered setae; distomedial margin with a row of small, short, feathered setae.

***Right mandible*** (Fig. 5B–D). Canine with almost completely fused outer and inner incisors, incisors well developed, apically rounded; prostheca robust, apically with small denticles and comb-shaped structure; edge between mola and prostheca smooth, without setae; molar area with numerous small, apically rounded teeth; apex of mola with tuft of spines like setae.

***Left mandible*** (Fig. 5E–G). Canine with almost completely fused outer and inner incisors, well developed incisors, apically rounded; prostheca robust, apically with small denticles; margin between mola and prostheca smooth, without setae; molar area with numerous small, round teeth, apex of mola with tuft of thin setae.

***Maxilla*** (Fig. 5H). Short and compact; galea-lacinia (Fig. 5I) with long, robust, simple setae under crown. Inner dorsal row of setae with three denti-setae, distal denti-seta tooth-like, middle denti-seta slender, bifid and pectinate (Fig. 5J), proximal denti-seta slender, pectinate; innermost denticles with a row of robust, simple setae; medially with one seta and four short to long, simple setae. Short, stocky, 2-segmented maxillary palp, with scattered small setae; distal segment with distinct, small tip.

***Labium*** (Fig. 5K). Short and compact; glossa basally broad, narrower toward apex, slightly shorter than paraglossa; paraglossa sub-rectangular, broader than glossa, apically curved inward, apical margin with three rows of medium stout setae; labial palp 3-segmented, segment II with small distolateral expansion, segment III rounded, ventral surface covered with scattered setae.

***Hypopharynx*** (Fig. 5M). Lingua subequal to superlingua, apically rounded, with apical tuft of fine, long, simple setae; superlingua with distal margin slightly incurved, margin covered with fine simple setae.

***Thorax*. *Forewing pads*.** Highly developed related to body size; clearly divergent.

***Hindwing pads*.** Highly reduced.

**Figure 7.**
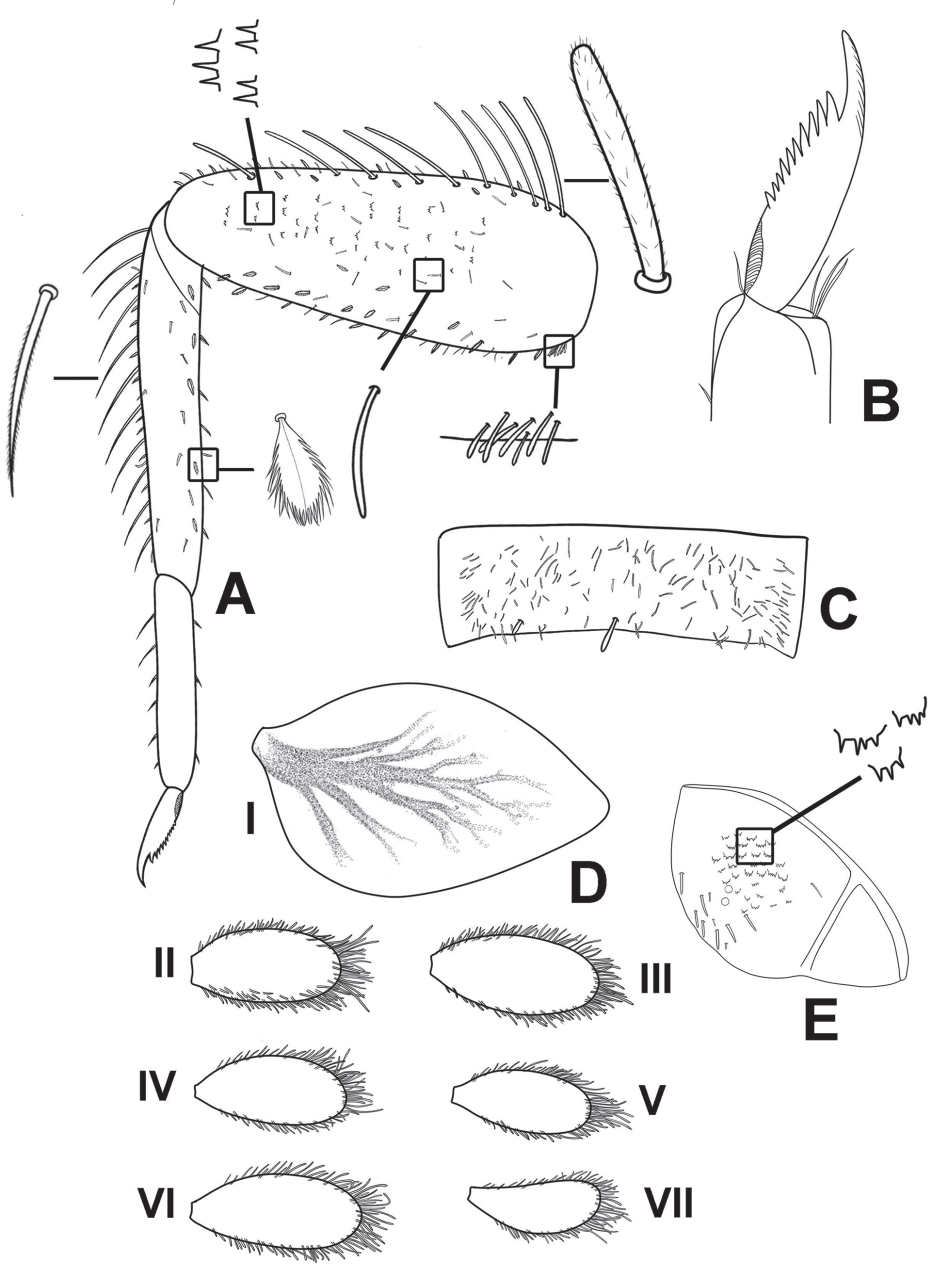
*Megabranchiellascutulata* sp. nov., larval morphology **A** foreleg **B** tarsal claw **C** tergite V **D** abdominal gills I–VII **E** Paraproct and notched scales on surface.

***Forelegs*** (Figs 7A, 12A). Dorsal margin of femur with a row of long, simple setae; short, stout, lanceolate, laterally pectinate setae and scattered fine hair-like setae along dorsal and ventral margins; femora patch present; dorsal surface with scattered tiny spine-like setae anteromedially; scattered long translucent scales present; dorsal margin of tibia with a row of long, simple setae; several broad, lanceolate, laterally pectinate setae and scattered hair-like setae along dorsal and ventral margins, patella-tibial suture present; tarsus dorsally with a row of spine-like simple setae, ventral margin bare or with a row of spine-like simple setae, surface covered with scattered fine hair-like setae; tarsal claw with one row of denticles increasing in length toward apex, subapical setae absent. ***Midlegs and hindlegs*.** As forelegs.

***Abdomen*** (Fig. 6B). ***Tergites*.** Posterior margin smooth, posterior marginal spines reduced to absent, tergal surface with scattered stout, fine, hair-like setae and scattered long translucent scales distally. Gonosyli under larval cuticule *Acentrella*-type (Fig. 6E).

***Gills*.** Seven pairs of gills present on abdominal tergites I–VII; gills I enlarged to covered abdominal sternites II–V, oriented ventrally (Fig. 6D), gill margin smooth, without fine hair-like setae; gills II–VII slightly oval, oriented dorsolaterally, gills margin smooth with scattered, long, fine hair-like setae.

***Paraproct*** (Fig. 7E). Margin smooth without marginal spines and without prolongation at posterior margin.

***Caudal filaments*.** Inner margin of cerci with very thin, long setae; median filament reduced shorter than 0.4× of cerci length, lateral margins with very thin, long setae.

###### Winged stage.

Unknown.

##### ﻿ Megabranchiella scutulata

Taxon classificationAnimaliaEphemeropteraEphemeroptera

Phlai-ngam & Tungpairojwong sp. nov.

6C4ABD25-9911-546D-9273-936C7A550A6C

https://zoobank.org/9DBB55FF-E63C-4DDA-9235-D825D0A0C08E

###### Materials examined.

***Holotype*.** Thailand, One male larva on slide (KKU-AIC), Chiang Mai, Mae On district, Mae Kampong, The Royal Project of Teen Tok, 18°52'01.65N, 99°19'20.83E, 779 m, 21.12.2020, S. Phlai-ngam leg.

***Paratypes*.** One larva on slide (KKU-AIC), same data as holotype; 21 larvae in alcohol, same data as holotype; Four larvae in alcohol (MZL), same data as holotype.

###### Other materials.

Two larvae in alcohol (ZMKU), Thailand, Chiang Rai, Muang district, Pong Phra Baht waterfall, 20°00'39.60N, 99°48'14.47E, 476 m, B. Boonsoong and C. Sutthinun leg.

###### Description.

***Coloration*** (Figs 1–2). Head dorsally yellow to brownish, with a darker brown pattern between ocelli. Thorax dorsally brown; pronotum with dark brown pattern laterodorsally, mesonotum with longitudinally dark brown pattern medially. Abdomen dorsally brownish; tergite I light brown; tergites I–VIII brown with reddish brown pattern posterolaterally, tergites IV and V slightly lighter; tergites IX and X light brown with or without pale markings. Head and thorax ventrally light brown to yellow; abdomen ventrally light brown; sternites I–V light brown; sternites V–X medium to dark brown (Fig. 3). Legs light brown; dorsal, ventral, and apical femur margins dark brown; claws distally dark brown. Caudal filaments brownish.

***Body*** (Fig. 4). Relatively short and ventrally flattened (Fig. 4B), body length 3.6 mm, covered with scattered long, hair-like setae.

***Head*** (Fig. 4B). Lateral view rounded, head width ca. 1.5 × as long as head length.

***Antenna*.** ca. 2 × as long as head length (Fig. 4B); scape without process, subequal in width and length, pedicel length, ca. 2 × as long as width, scape and pedicel almost bare, without scales bases and spines, covered with scattered long fine setae; flagellum covered with scattered long fine setae in each segment.

***Labrum*** (Fig. 5A). Broadly rounded; wider than long, width ca. 1.75 × as long as length; dorsal surface with submarginal row composed of one long, point, simple seta medially plus three medium, simple setae anterolaterally, dorsal surface with scattered simple, hairlike setae; distal margin with shallow anteromedian notch. Ventrally with submarginal row of setae composed of lateral and anterolateral long, feathered setae and medial long, pectinate setae; ventral surface with six short, spine-like setae near lateral and anterolateral margin.

***Right mandible*** (Fig. 5B). Canine with 4 + 4 apically rounded denticles (Fig. 5C), largely fused outer and inner incisors, inner margin of inner incisor with small denticulation; prostheca robust (Fig. 5D), apically with small denticles and comb-shaped structure; margin between mola and prostheca smooth, without setae; mola with well-developed denticulation; apex of mola with tuft of spines like setae.

***Left mandible*** (Fig. 5E). Canine with 4 + 3 apically rounded denticles (Fig. 5F), largely fused outer and inner incisors, outer and inner incisors separated by a small, rounded tooth; prostheca robust (Fig. 5G), apically with small denticles and comb-shaped structure; margin between mola and prostheca smooth, without setae; mola with reduced denticulation, apex of mola with tuft of spines like setae.

***Maxilla*** (Fig. 5H). Short and compact; galea-lacinia (Fig. 5I) with long, robust, simple seta under crown; inner dorsal row of setae with three denti-setae, distal denti-seta tooth-like, middle denti-seta slender, bifid and pectinate (Fig. 5J); proximal denti-seta slender, pectinate; inner ventral row of seven robust, simple setae; medially with one seta and four short to long, simple setae. Maxillary palp 2-segmented, with scattered small, blunt setae; distal segment with distinct, small tip at apex.

***Labium*** (Fig. 5K). Glossa basally broad, narrower toward apex, slightly shorter than paraglossa; inner margin with medium, pointed, simple setae; apex with four long, robust, pectinate setae; basal area with fine scattered setae. Paraglossa sub-rectangular, broader than glossa, apically curved inward, apical margin with three rows of long, robust, apically pectinate setae (Fig. 5L), ventrally with 4–5 long, spine-like setae near inner margin, with an arch of 4–5 long, simple setae on outer margin; basal area with a single medium seta. Labial palp 3-segmented, segment I rectangular and broad, covered with scattered fine, setae and several micropores; segment II with small distolateral expansion, with a few scattered, simple setae and row of setae reduced to two large, blunt, robust, simple setae near distal margin; segment III rounded, covered with long, robust, simple setae.

***Hypopharynx*** (Fig. 5M). Lingua subequal to superlingua, apically rounded, with apical tuft of fine, simple setae; superlingua with distal margin slightly incurved, margin covered with fine simple setae.

**Figure 8.**
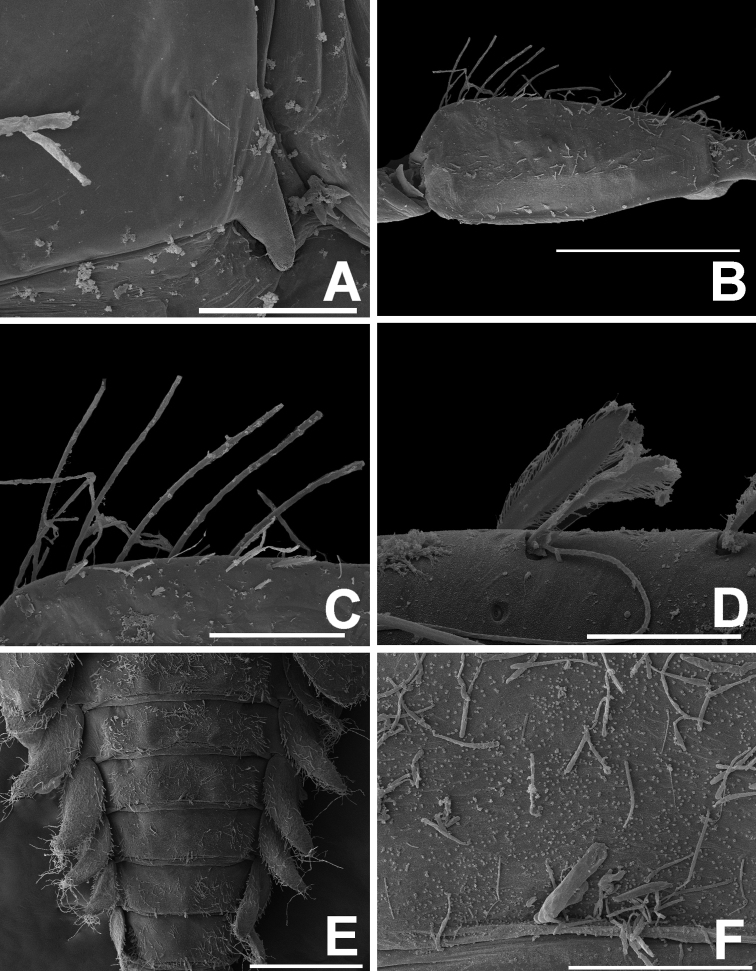
*Megabranchiellascutulata* sp. nov., larval morphology (SEM) **A** hindwing pad **B** forefemur (dorsal view) **C** setae on dorsal margin of forefemur **D** setae on ventral margin of forefemur **E** abdominal tergites **F** tergite V. Scale bars: 50 um (**A**); 100 um (**B–C, F**); 20 um (**D**); 500 um (**E**).

***Thorax*. *Hindwing pads*** (Fig. 8A). Highly reduced.

***Forelegs*** (Fig. 7A). Ratio of foreleg segments 0.58: 0.48: 0.22. *Femur*. Length ca. 2.5× maximum width. Dorsal margin of femur (Figs 6A, 8B–C) with a row of 11–13 long, robust, apically rounded, simple setae; short stout, lanceolate, laterally pectinate setae and scattered fine hair-like setae along dorsal and ventral margins (Fig. 8D); femora patch present; surface with scattered tiny spines anteromedially (Fig. 8E); scattered long translucent scales present; dorsal margin of tibia with a row of long, simple setae; several short stout, lanceolate, laterally pectinate setae and scattered hair-like setae along dorsal and ventral margins, patella-tibial suture present; tarsus dorsally with a row of fine, spine-like, simple setae, ventral margin bare or with a row of fine, spine-like, simple setae, surface covered with scattered fine hair-like setae; tarsal claw (Fig. 7B) with one row of about 12 denticles increasing in length toward apex, subapical setae absent. ***Midlegs and hindlegs***. As forelegs.

***Abdomen*** (Fig. 6B). ***Tergites*.** Posterior margin smooth, posterior marginal spines extremely reduced to absent, tergal surface with scattered stout, fine, hair-like setae and scattered long translucent scales distally (Figs 6C, 7C, 8F); abdominal sternites without posterior marginal spines; sternal surface with loose scattered fine, hair-like setae.

***Gills*** (Fig. 7D). Seven pairs of gills present on abdominal tergites I–VII; gills I (Fig. 6D) enlarged to covered abdominal sternites II–V, ventrally oriented, relatively rhombus shape, length approximately 1.4× of width, medially part broad, tracheation extending from main trunk to outer margin, gill margin smooth, surface and gill margin without long, fine hair-like setae; gills II–VII oriented dorsolaterally, slightly oval length approximately 2.1× of width, gill margin smooth, surface and gill margin covered with scattered long, fine hair-like setae.

***Gonostyli bud*** (Fig. 6E). *Acentrella*-type, three-segmented, segment I very short, 0.3 × of segment II length, segment III relatively short and broad, rounded at apex.

***Paraproct*** (Fig. 7E). Margin smooth without marginal spines and without prolongation at posterior margin. Surface without scale bases, with micropores and fine, stout, simple setae and scattered fine, hair-like setae, and with a patch of notch scales.

***Caudal filaments*.** Cerci 0.4× of body length, inner margin of cerci with very thin, long setae; median filament 0.5 × of cerci length, lateral margins with very thin, long setae.

###### Winged stages.

Unknown

###### Etymology.

The name of the species “scutula” refers to the outline of abdominal gill I which is rhombus- shaped.

###### Distribution.

Northern part of Thailand (Chiang Mai and Chiang Rai Provinces) (Fig. 15).

###### Ecological notes.

The larvae were collected in headwater stream (Mae Kampong River) (Fig. 14A) and Pong Phra Baht waterfall (Fig. 14B). The sampling sites were located with altitudes of 475–780 m a.s.l. Both stream and waterfall were situated in forest areas with relatively complete canopy cover on mountains in the northern part of Thailand. The stream was in The Royal Project of Teen Tok area which has some human disturbances resulting from touristic attractions and resorts. The substrates were dominated by 50% cobbles, 20% pebble, 20% boulders, 10% gravels respectively with sand bottom. The waterfall was located upstream. The larvae were found on the surface of cobbles in fast-flowing water (Fig. 14C).

##### ﻿ Megabranchiella longusa

Taxon classificationAnimaliaEphemeropteraEphemeroptera

Phlai-ngam & Tungpairojwong sp. nov.

31CA90DC-3320-585B-8D2A-2FC947835621

https://zoobank.org/65816410-4304-459F-85AF-5C372989F48A

###### Holotype.

One male larva on slide and SEM stubs (KKU-AIC), Thailand, Chiang Mai, Chom Thong district, Ban Luang, Siribhum waterfall, 18°32'50.02N, 98°30'49.79E, 1,359 m, 11.03.2021, B. Boonsoong and C. Sutthinun leg.

###### Paratype.

One larva in alcohol (ZMKU), same data as holotype.

###### Other materials.

One larva in alcohol (MZL), Thailand, Nan, Bo Kluea district, Kluea district Tai, Sapan River, 19°09'19.09N, 101°10'12.96E, 995 m, B. Boonsoong and C. Suttinun leg.

###### Description.

**Figure 9.**
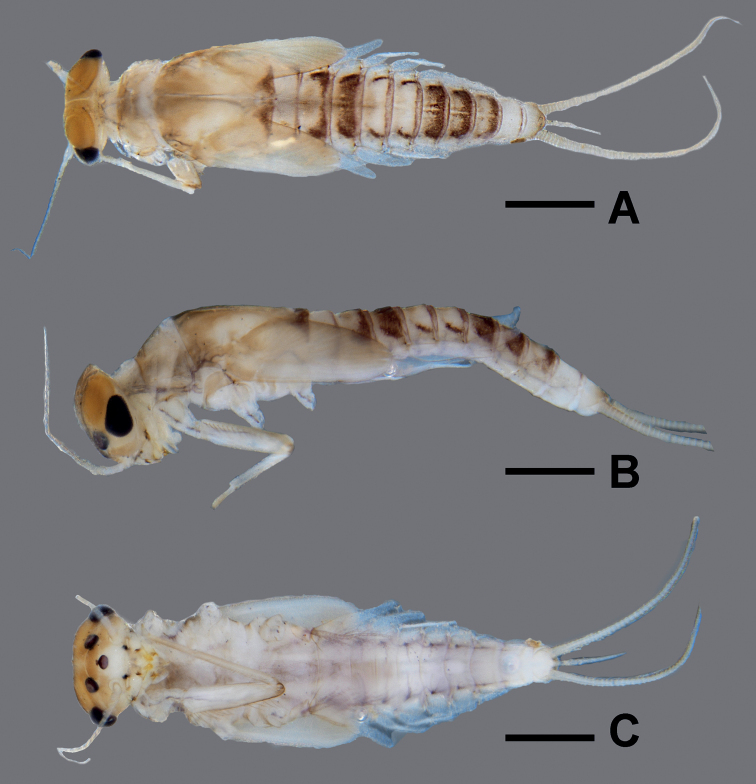
*Megabranchiellalongusa* sp. nov., larval habitus: **A** dorsal view **B** lateral view **C** ventral view. Scale bars: 1 mm.

***Coloration*** (Fig. 9A–B). Head dorsally brownish, darker brown along frontal suture. Thorax dorsally brown; pronotum with dark brown pattern medially, mesonotum with longitudinally darker brown pattern medially. Abdomen light brown with dark brown pattern; tergites I–VIII brownish, with darker brown marks laterally to posterior margin, tergites II–III with a paired of pale dots medially, tergites IV–VIII with distinct paired of pale, oblique streak, and with a pale, longitudinal pattern medially, tergites IX–X paler than other tergites, with the same pattern as tergites IV–VIII; abdomen ventrally light brown (Fig. 9C). Legs light brown; dorsal, ventral, and apical femur margins dark brown; claws distally dark brown. Caudal filaments brownish.

***Body*** (Fig. 9). Ventrally flattened (Fig. 9C), body length 3.5 mm, covered with scattered long, hair-like setae.

***Head*** (Fig. 9B). Lateral view rounded, head width ca. 1.2 × as long as head length.

***Antenna*.** Ca. 2 × as long as head length (Fig. 9B); scape without process, subequal in width and length, slightly shorter than pedicel, pedicel ca. 2 × as long as width, scape and pedicel almost bare, without scales bases, covered with scattered long, fine setae; flagellum covered with scattered long, fine setae.

**Figure 10.**
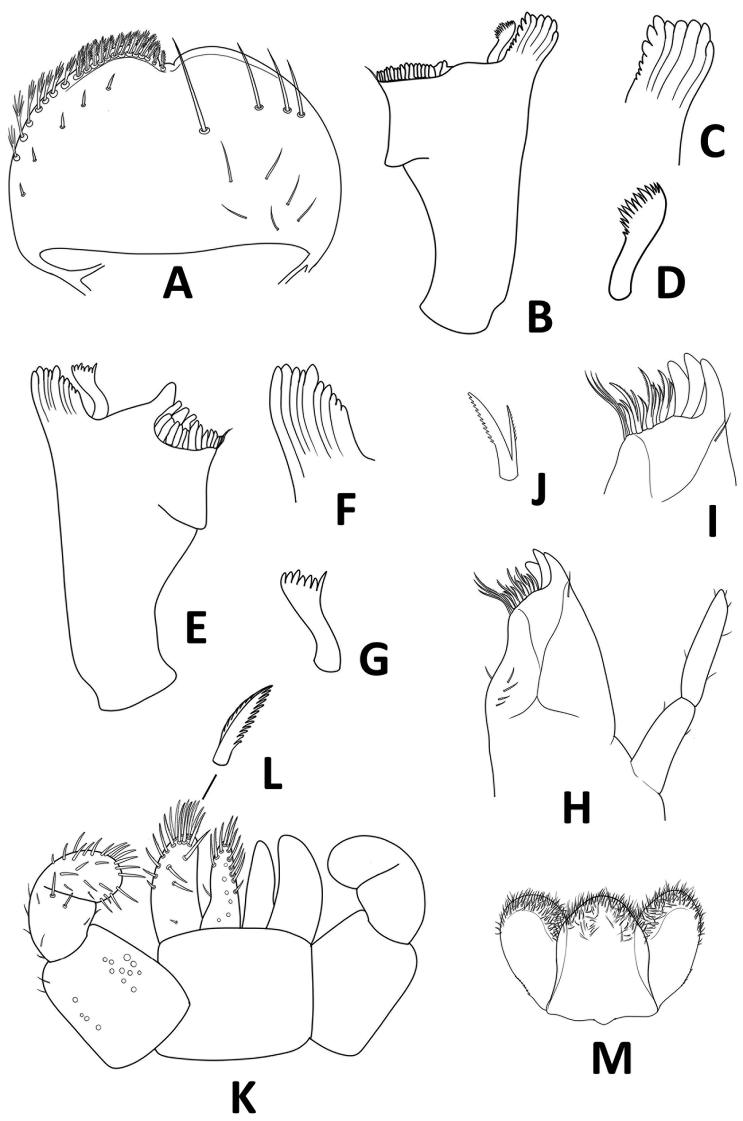
*Megabranchiellalongusa* sp. nov., larval morphology **A** labrum **B** right mandible **C** right incisor **D** right prostheca **E** left mandible **F** left incisor **G** left prostheca **H** maxilla **I** apex of galea-lacinia **J** denti-seta **K** labium **L** long, robust, pectinate setae **M** hypopharynx.

***Labrum*** (Fig. 10A). Broadly rounded; wider than long, width ca. 1.17 × as long as length; dorsal surface with submarginal row composed of one long, point, simple seta medially plus three long, point, simple setae anterolaterally, dorsal surface with scattered simple, hairlike setae; distal margin with anteromedian notch shallow. Ventrally with submarginal row of setae composed of lateral and anterolateral long, feathered setae and medial long, pectinate setae; ventral surface with five short, spine-like setae near lateral and anterolateral margin.

***Right mandible*** (Fig. 10B). Canine (Fig. 10C) with 4 + 4 apically rounded denticles, largely fused outer and inner incisors; inner margin of inner incisor with small denticulation; prostheca robust (Fig. 10D), apically with small denticles and comb-shaped structure; mola between mola and prostheca smooth, without setae; mola with well-developed denticulation; apex of mola with tuft of spines like setae.

***Left mandible*** (Fig. 10E). Canine (Fig. 10F) with 4 + 4 apically rounded denticles, largely fused outer and inner incisors; inner margin of inner incisor with small denticulation; prostheca robust, apically with small denticles and comb-shaped structure (Fig. 10G); mola between mola and prostheca smooth, without setae; mola with reduced denticulation, molar area with numerous small, round teeth, apex of mola with tuft of spines like setae.

***Maxilla*** (Fig. 10H). Short and compact; galea-lacinia (Fig. 10I) with long, robust, simple setae under crown; inner dorsal row of setae with denti-setae; distal denti-seta tooth-like, middle denti-seta (Fig. 10J) slender, bifid and pectinate; proximal denti-seta slender, pectinate; inner ventral row of six robust, simple setae; medially with one seta and four simple setae. Maxillary palp 2-segmented, with scattered small, blunt setae; distal segment with distinct, small tip at apex.

***Labium*** (Fig. 10K). Short and compact; glossa basally broad, narrower toward apex, slightly shorter than paraglossa; inner margin with medium, pointed, simple setae; apex with four long, robust, pectinate setae. Paraglossa sub-rectangular, broader than glossa, apically curved inward, apical margin with three rows of long, robust, apically pectinate setae (Fig. 10L), ventrally with 2–3 long, spine-like setae near inner margin, with an arch of 4–5 long, simple setae on outer margin. Labial palp 3-segmented, segment I rectangular and broad, covered with scattered fine, setae and several micropores; segment II with small distolateral expansion, with a few scattered, simple setae and a row of setae reduced to two large, blunt, robust, simple setae near distal margin; segment III rounded, covered with long, robust, simple setae.

***Hypopharynx*** (Fig. 10M). Lingua subequal to superlingua, apically rounded, with apical tuft of fine, short simple setae; superlingua with distal margin rounded, with fine, short simple setae along margin.

***Thorax*. *Hindwing pads*** (Fig. 13A). Highly reduced.

**Figure 11.**
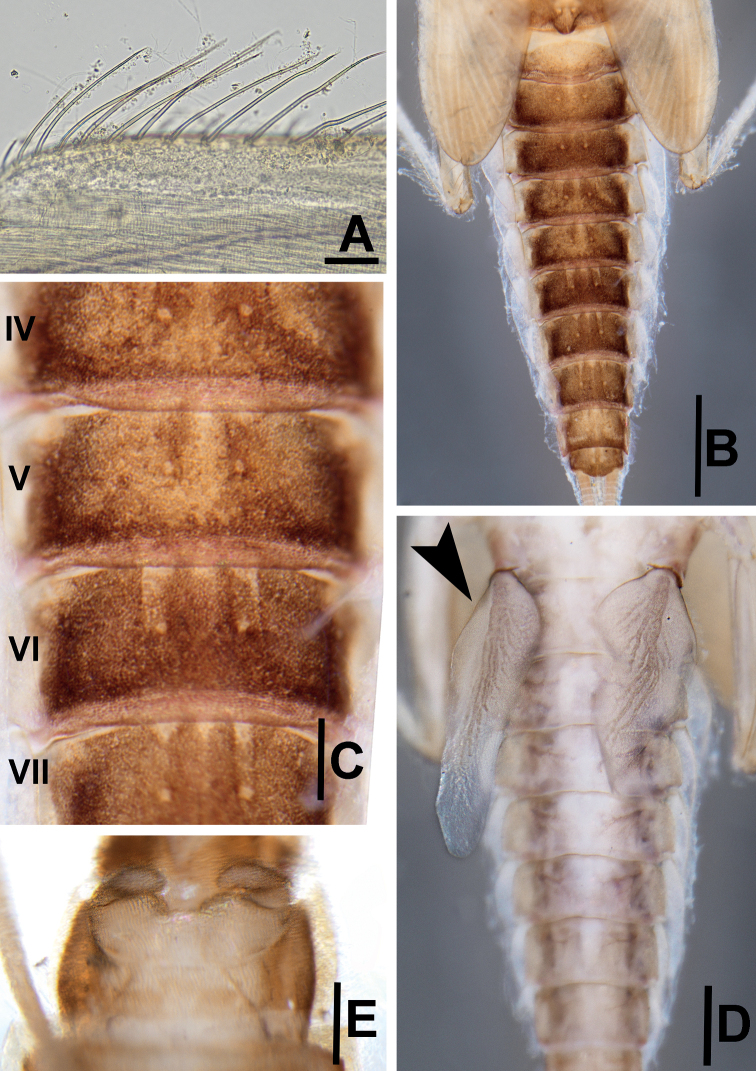
*Megabranchiellalongusa* sp. nov., larval morphology **A** dorsal margin of femur **B** dorsal view of abdomen **C** enlargement of abdominal tergites IV–VII **D** ventral view of abdominal gills I (arrow) **E** ventral view of gonostyli. Scale bars: 0.1 mm (**A**); 0.8 mm (**B**); 0.2 mm (**C**); 0.4 mm (**D**); 0.3 mm (**E**).

**Figure 12.**
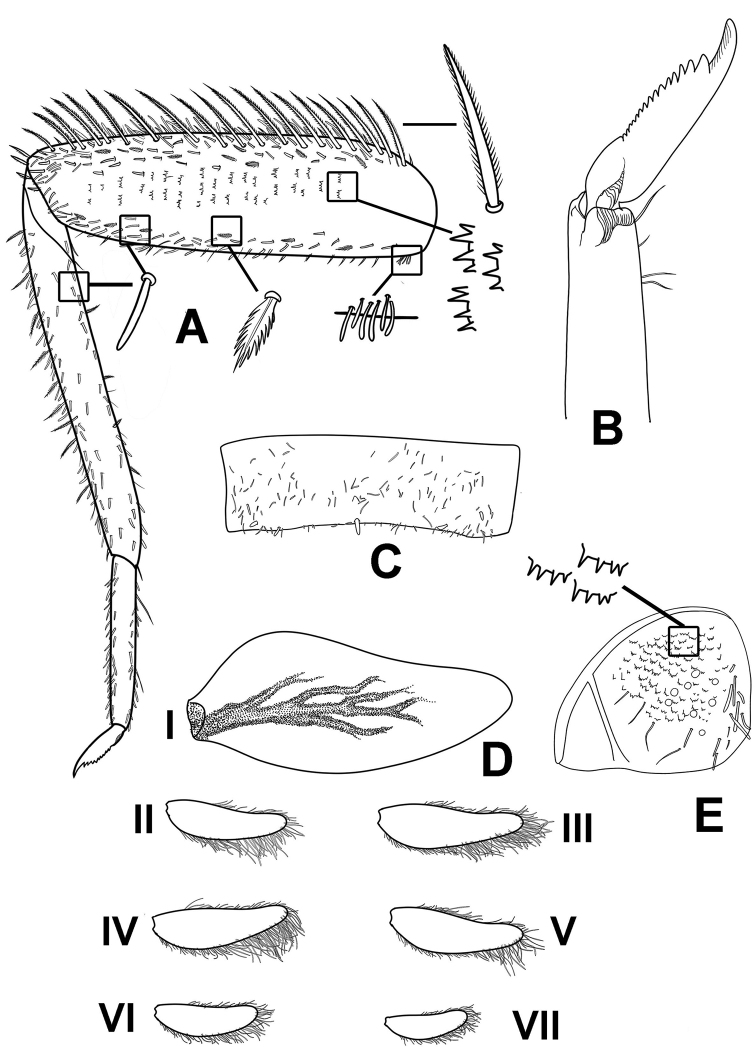
*Megabranchiellalongusa* sp. nov., larval morphology **A** foreleg **B** tarsal claw **C** tergite V **D** abdominal gills I–VII **E** paraproct and notched scales on surface.

**Figure 13.**
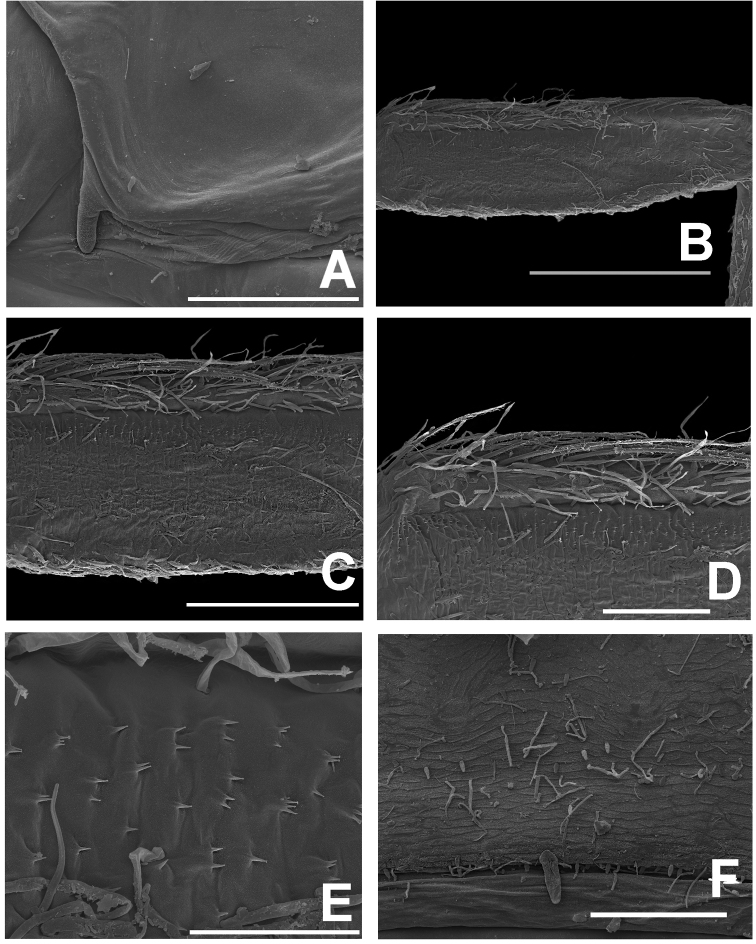
*Megabranchiellalongusa* sp. nov., larval morphology (SEM) **A** hindwing pad **B** forefemur (dorsal view) **C–D** setae on dorsal margin of forefemur **E** spines on dorsal surface of forefemur **F** tergite V. Scale bars: 100 um (**A, D, F**); 400 um (**B**); 200 um (**C**); 30 um (**E**).

***Forelegs*** (Fig. 12A). Ratio of foreleg segments 0.68: 0.59: 0.25. *Femur* (Fig. 13B). Length ca. 3× maximum width. Dorsal margin of femur (Figs 11A, 13C–D) with a row of 18–20 long, robust, apically pointed, laterally pectinated setae; short stout, lanceolate, laterally pectinate setae and scattered fine hair-like setae along dorsal and ventral margins; femora patch present; surface with scattered tiny spines anteromedially (Fig. 13E); dense long, fine, small apically blunt, hair-like setae present; dorsal margin of tibia with a row of long, apically pointed, pectinate setae; several short stout, lanceolate, laterally pectinate setae and scattered short, apically pointed, hair-like setae along dorsal and ventral margins, patella-tibial suture present; tarsus dorsally with a row of fine, simple setae; ventral margin with a row of fine, simple setae; surface covered with scattered fine hair-like setae. Tarsal claw (Fig. 12B) with one row of about 16 denticles increasing in length toward apex, subapical setae absent. ***Midlegs and hindlegs*.** As forelegs.

***Abdomen*** (Fig. 11B). ***Tergites*.** Posterior margin smooth, posterior marginal spines extremely reduced to absent (Figs 11C, 12C, 13F), tergal surface with scattered fine, hair-like setae and scattered long translucent scales distally; abdominal sternites without posterior marginal spines, sternal surface with loose scattered, fine, hair-like setae.

***Gills*** (Fig. 12D). Seven pairs of gills present on abdominal tergites I–VII, slender and elongated; gills I (Fig. 11D) enlarged to covered abdominal sternites II–V, oriented ventrally, relatively elongated shape with length approximately 2.5× of width, medially part broad, tracheation extending from main trunk and outer margin, gill margin smooth, surface and gill margin without long, fine hair-like setae; gills II–VII oriented dorsolaterally, slightly oval and slender with length approximately 3.3× of width, gill margin smooth, surface and gill margin covered with scattered long, fine hair-like setae.

***Gonostyli bud*** (Fig. 11E). Acentrella-type, three-segmented, segment I very short, 0.3 × of segment II length, segment III relatively short and broad, rounded at apex.

***Paraproct*** (Fig. 12E). Margin smooth without marginal spines and without prolongation at posterior margin, surface without scale bases, with micropores and fine, stout, simple setae and scattered fine, hair-like setae, and with a patch of notch scales.

***Caudal filaments*.** Cerci 0.4× of body length, inner margin of cerci with very thin, long setae; median filament 0.5 × of cerci length, lateral margins with very thin, long setae.

###### Winged stage.

Unknown.

###### Etymology.

The name of the species “longusa” refers to the outline of abdominal gill I which is elongate- shaped.

**Figure 14.**
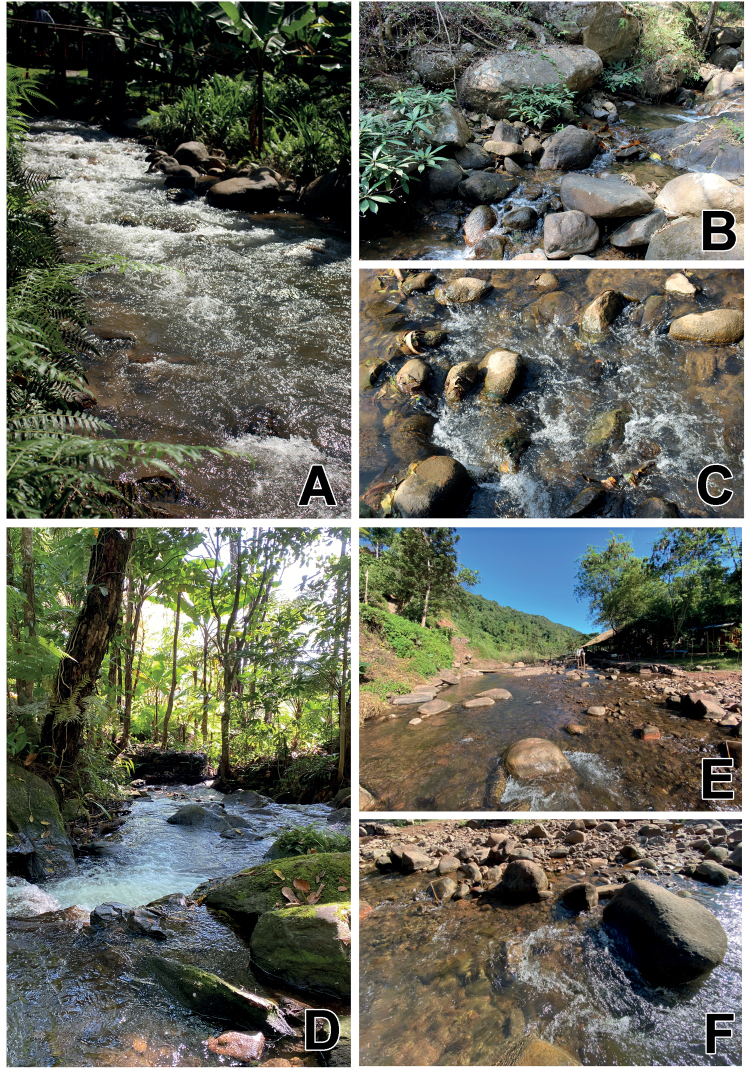
Type locality and larval habitats of *Megabranchiella* gen. nov. **A–C** type locality and larval habitats of *M.scutulata* sp. nov. **A** Mae Kampong stream, The Royal Project of Teen Tok, Chiang Mai Province **B** Pong Phra Baht Waterfall, Chiang Rai Province **C** fast-flowing water with pebble and cobble habitats (**D–F**) type locality and larval habitats of *M.longusa* sp. nov. **D** Siribhum waterfall, Chiang Mai Province **E** Sapan River, Bor Kluea district, Nan Province **F** fast-flowing water with boulder, cobble, and pebble habitats.

###### Distribution.

**Figure 15.**
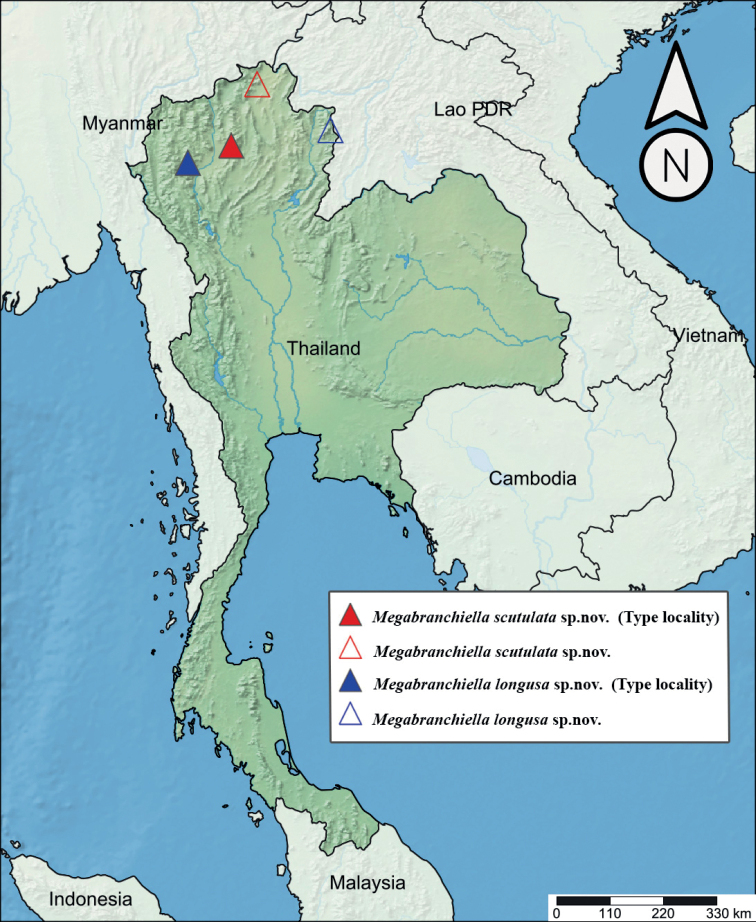
Distribution of genus *Megabranchiella* gen. nov. in Thailand **A** distribution of *M.scutulata* sp. nov. (red triangle) **B** distribution of *M.longusa* sp. nov. (blue triangle).

Northern part of Thailand (Chiang Mai and Nan Provinces) (Fig. 15).

###### Ecological notes.

The larvae were collected in Siribhum waterfall (Fig. 14D) and headwater stream (Sapan River River) (Fig. 14E). The sampling sites were located at high altitudes of 995–1,360 m a.s.l. in forest areas on mountains in the northern part of Thailand. The waterfall was in the upper stream of the Ping River, and the substrate types were dominated by boulders, cobbles, pebbles, gravel, and a sand bottom. The stream was in the Sapan River and located near the resort which can be disturbed by touristic attractions. The larvae were found on the surface and underside of cobbles in fast-flowing water (Fig. 14F).

## ﻿Discussions

### ﻿Generic affinities

The morphological characters of *Megabranchiella* gen. nov. clearly confirm that it belongs to the family Baetidae. These include a Y-shaped epicranial suture reaching ventrally of the lateral ocelli, relatively long antennae originating anterolaterally on the head, the developing turbinate eyes of late instar male larvae, a labrum with a median incision, the shape of the glossa, widen basally, and the shape of the right and left prostheca of the mandibles. This genus can be assigned to the clade Anteropatellata due to the presence of a patella-tibial suture on the foreleg and referred to Baetofemorata. The taxon Baetofemorata or Baetis/fg8 sensu Kluge & Novikova, 2011 includes the lineages Baetinae and Acentrella/fg1. As other members of this taxon, *Megabranchiella* gen. nov. is characterized by a ventral femoral patch (villopore) on the fore femur of larva.

Despite the presence of this femoral patch, which has been used to assign taxonomic status, the position within Baetofemorata remains unclear. Taxon Acentrella/fg1 is a mayfly group assigned to the Baetofemorata. This taxon is composed of the genera *Acentrella* Bengtsson, 1912, *Acerobiella* Gattolliat, 2012, *Asiobaetodes* Gattolliat, 2012, *Jubabaetis* Müller - Liebenau, 1980, *Liebebiella*, and *Platybaetis* Müller-Liebenau, 1980 and *Tanzaniops* McCafferty & Barber-James, 2005 (Kluge and Novikova 2011). The *Megabranchiella* gen. nov. larvae seem intimately related to Acentrella/fg1 by the following larval characters: (i) labial palp 2^nd^ segment without large inner-apical projection, 3^rd^ segment widened and rounded; (ii) short body, with ventrally flattened, thorax enlarged, abdomen diminished; (iii) head hypognathous, shortened or compact mouthparts; (iv) leg bases widely separated, femur with a row of long, dense setae along dorsal margin, tibia with a similar setal row; and (v) cerci long, with reduced paracercus (Sroka and Arnekleiv 2010; Kluge and Novikova 2011). Therefore, this study assigned this new genus to Acentrella/fg1 Kluge & Novikova, 2011, along with two closely related genera of Acentrella/fg1: *Acentrella* (Acentrella/fg1 Kluge & Novikova, 2011) and *Liebebiella* (Liebebiella/g1 Kluge & Novikova, 2011).

*Megabranchiella* gen. nov. has a ventrally flattened body with compact mouthparts, as in *Acentrella* (Bengtsson, 1912) and *Liebebiella* (Waltz & McCafferty, 1987). The labial palps have a weakly developed inner lobe with a rounded terminal segment. The maxillary palps are 2-segmented, while the 2^nd^ segment with the apical tip is distinct in *Megabranchiella* gen. nov. The hind wing pads are reduced to absent in all genera (Waltz and McCafferty 1987; Kluge and Novikova 2011). The dorsal margin of the femur has a regular row of long, dense setae and distinct multiciliate setae in *Liebebiella*. The dorsal margin of the tibia has two rows of relatively long, dense, multiciliate setae in *Liebebiella*, while it can be present with 1 or 2 rows in *Acentrella* (Waltz and McCafferty 1987; Kluge et al. 2013). *Megabranchiella* gen. nov. has only one row of regular, long, simple setae on the dorsal margin of the tibia. The preapical tarsal seta is highly developed in *Liebebiella* but absent in *Acentrella* and *Megabranchiella* gen. nov. The subapical setae of the tarsal claw show are present in some species of *Acentrella* (Waltz and McCafferty, 1987; Kluge et al. 2013) and absent in *Megabranchiella* gen. nov. Seven pairs of abdominal gills are present in *Acentrella* and *Liebebiella*; these gills are simple, dorsally oriented, and smooth margined, with scattered fine, hair-like setae. The genus *Megabranchiella* gen. nov. can be distinguished from other genera by its gill I ventrally oriented, enlarged to cover abdominal sternites II–V, and the gill margins are smooth, with scattered fine, hair-like setae. Gills II–V are dorsally oriented and have smooth margins with long scattered fine, hair-like setae. The posterior marginal spines of the abdomen are varied and poorly developed in *Acentrella* and *Megabranchiella* gen. nov. but are distinct in *Liebebiella*. The gonostyli buds of these genera are of *Acentrella* type; the paracercus is reduced or vestigial (Table 2).

**Table 2.** Larval character comparisons of *Megabranchiella* gen. nov. and related genera.

Characters	*Acentrella*	*Liebebiella*	*Megabranchiella* gen. nov.
**Body**	Body ventrally flattened	Body ventrally flattened	Body ventrally flattened
**Mouthpart**	Compact, the 2^nd^ segment of labial palp with weakly developed inner lobe, the 3^rd^ segment evenly rounded to slightly truncate	Compact, the 2^nd^ segment of labial palp with weakly developed inner lobe, the 3^rd^ segment rounded, weakly tapered, or flattened apically	Compact, the 2^nd^ segment of labial palp with weakly developed inner lobe, the 3^rd^ segment relatively rounded
**Maxillary palp**	2-segmented, terminal segment with or without apical tip	2-segmented, terminal segment usually with apical tip	2-segmented, terminal segment with apical tip
**Hindwing pad**	Present, reduced or absent	Absent	Highly reduced
**Femur**	Dorsal margin of femur usually with a row of relatively long setae	Dorsal margin of femur with relatively long, dense multiciliate setae	Dorsal margin of femur with a regular row of long, simple setae
**Ventral femoral patch**	Present	Present	Present
**Tibia**	Dorsal margin of tibia with 1 or 2 regular rows of long, simple setae	Dorsal margin of tibia usually with 1 or 2 rows of dense, long, multiciliate setae	Dorsal margin of tibia with a row of long, simple setae
**Tarsus**	Without a preapical seta	With a preapical seta	Without a preapical seta
**Tarsal claw**	Generally without subapical setae (presence in a few species)	Without subapical setae	Without subapical setae
**Abdominal gills**	Usually 7 pairs (8 pairs in *A.joosti*), dorsally oriented; gill margin smooth with a few scattered fine hair-like setae	Seven pairs, dorsally oriented; gill margin smooth with a few scattered fine hair-like setae	Seven pairs; gill I enlarged to cover sternites II–V, ventrally oriented, gill margin smooth without dense long fine hair-like setae; gill II–VII dorsally oriented, gill margin with dense long fine hair-like setae
**Posterior marginal spines**	Poorly developed, often spiculate or multidentate	Well developed	Reduced to absent
**Paracercus**	Generally reduced (sometimes to a segment)	Reduced to a few segments (generally 10–14 segments)	Reduced to a few segments (Approximately 12–15 segments)
**Distribution**	Palearctic, Nearctic and Oriental realms	Oriental realm	Northern Thailand (Chiang Mai, Chiang Rai and Nan Provinces)
**References**	Waltz and McCafferty (1987); Sroka and Arnekleiv (2010); Kluge and Novikova (2011)	Waltz and McCafferty (1987); Kluge and Novikova (2011); Kluge et al. (2013)	Present study

### ﻿Abdominal gill segment I

The gills allow a straightforward identification of the genus: the position of the larval abdominal gills of *Megabranchiella* gen. nov., specifically the ventral orientation of gill I, is an important character. Ventrally oriented gills are also present in the genera *Afrobaetodes* (Demoulin, 1970) and *Baetodes* (Needham & Murphy, 1924), but they differ from *Megabranchiella* by the following features: the number of ventrally oriented gills, the number of rows of denticles on the tarsal claws, and the presence of accessories’ gills (Gattolliat and Sartori 1999). *Megabranchiella* gen. nov. cannot be assigned to the same group as these genera. The genus *Cymbalcloeon* also has ventrally oriented gills but is defined to a non-Baetovectata taxon, apart from *Afrobaetodes* and *Baetodes*. Even though *Cymbalcloeon* and *Megabranchiella* have ventrally inserted gills, morphological characters assign them to different lineages. *Cymbalcloeon* has strong characters such as absence of gills on segments I–IV, and modified gills on abdominal segments V–VII (Suttinun et al. 2020). The genus *Asiobaetodes* Gattolliat, 2012 also possesses ventrally oriented gills; moreover this genus belongs to Acentrella/fg1. *Megabranchiella* and *Asiobaetodes* can be easily separated by the position of the gills II to VII and the presence/absence of a subapical setae on the ventral margin of tarsi (Gattolliat 2012).

### ﻿Larval body and movement

The larval body of *Megabranchiella* gen. nov. was comparable to Acentrella/g1 (including *Acentrella* and *Liebebiella*), as the larvae are adapted for inhabitance on stones in rapid flowing water. *Megabranchiella* gen. nov. larvae have the same general *Acentrella*-type body, which is dorso-ventrally flattened. They have lost the primary siphlonuroid swimming specialization and lost the ability for normal swimming, similarly to Acentrella/g1 larvae. The legs of Acentrella/g1 and *Megabranchiella* gen. nov. larvae are widely separated and cannot be stretched backward and pressed to the body. The abdomen is too short, with relatively long cerci, so the larvae cannot make undulating movements and cannot serve as a swimming flipper. In the natural conditions, they do not swim but crawl on the stone surfaces. When the larvae try to swim, they make poorly effective dorso-ventral movements with the abdomen. They swim upward, then stop and slowly fall passively, with their legs kept in lateral position, the abdomen bent dorsally, and the cerci diverging (see Kluge and Novikova 2011 for detailed description of swimming movements in other genera of Acentrella/g1).

### ﻿Species diversity of *Megabranchiella*

Surprisingly, two species of this new genus were found in the northern part of Thailand. *Megabranchiellascutulata* nov. sp. was found in Chiang Mai and Chiang Rai provinces, while *Megabranchiellalongusa* sp. nov. was collected in Chiang Mai and Nan provinces. These two *Megabranchiella* larvae can be separated by the following diagnostic characters: i) the dorsal pattern of the body; ii) the number of spine-like setae near the lateral and anterolateral margins of labrum; iii) the number of canines of the mandible; iv) the shape of the setae on the dorsal margin of the femur; these setae are long, apically rounded, simple in *Megabranchiellascutulata* nov. sp. while they are dense long, robust, apically pointed, pectinate in *Megabranchiellalongusa* sp. nov., vi) number of denticles of the tarsal claw, and vii) the shape of the abdominal gills, which are more elongated and slenderer in *Megabranchiellalongusa* sp. nov. (Table 3).

**Table 3.** Larval character comparisons of two new species of *Megabranchiella* gen. nov.

**Characters**		***M.scutulata* sp. nov.**	***M.longusa* sp. nov.**
**Hindwing pads**		Highly reduced	Highly reduced
**Femur**	Dorsal margin	Regular row of long, apically rounded, simple setae	Regular row of dense long, robust, apically pointed, pectinate setae
	Number of dorsal setae	11–13	18–20
	Dorsal surface	with scattered tiny spines and some long, apically blunt, fine, hair-like setae	with scattered tiny spines and dense long, apically blunt, fine, hair-like setae
**Tibia**	Dorsal margin	A regular row of long, apically pointed, pectinate setae	A regular row of long, apically pointed, pectinate setae
**Abdominal gills**	Gill I	Enlarged to covered abdominal sternites II–V, oriented ventrally, relatively rhombus shape, gill margin smooth	Enlarged to covered abdominal sternites II–V, oriented ventrally, relatively rhombus shape, gill margin smooth
	Length/width gill I	1.4×	2.5×
	Gill II–VII	Oriented dorsally, slightly oval, gill margin smooth, surface and gill margin covered with scattered long, fine hair-like setae	Oriented dorsally, slightly oval and slender, gill margin smooth, surface and gill margin covered with scattered long, fine hair-like setae
	Length/width gill II–VII	2.1×	3.3×
**Gonostyli bud**		*Acentrella*-type, 3-segmented	*Acentrella*-type, 3-segmented,
**Terminal filament**	Paracercus	Reduced to several segments; approximately 0.4× cerci length	Reduced to several segments; approximately 0.3× cerci length
**Distribution**		Northern Thailand (Chiang Mai and Chiang Rai Provinces)	Northern Thailand (Chiang Mai and Nan Provinces)

### ﻿Ecological notes and adaptation

*Megabranchiella* gen. nov. larvae were found in fast-flowing water in headwater streams and a waterfall. They live on stone surfaces of cobble and sand microhabitats in shallow water streams. Their habitat may possibly explain why *Megabranchiella* larvae have a ventrally oriented gill I. This feature might be important to *Megabranchiella* larvae for adaptation to fast-flowing shallow water streams. It is likely an adaptation to shallow fast streams as in *Afrobaetodes* and *Baetodes* (Gattolliat & Sartori, 1999). *Cymbalcloeon*, another genus with a ventral gill insertion, is also found in shallow streams, but occurs in slow-flowing water. Conceivably, ventral gill insertion could be a useful adaptation that allows mayflies to survive in shallow streams under various conditions.

The enlarged abdominal gill I is a remarkable morphological adaptation of *Megabranchiella* larvae. As in *Cymbalcloeon* larvae, a single pair of gills is enlarged. Because of the reductions of abdominal gills I–IV in *Cymbalcloeon*, this feature might be used for active respiration in slow-flowing water, whereas *Megabranchiella* larvae prefer fast-flowing water. The abdominal gills of mayflies have been revealed to perform various functions, such as respiration, osmoregulation, locomotion, water circulation, protection, and attachment (Zhou 2010; Kluge 2004). Why *Megabranchiella* larvae have modified their abdominal gill I to expand ventrally is unclear. However, considering their microhabitat and morphology (tracheation and margin), one possibility is that gill I can perform a role of attachment in addition to respiration. In fast-flowing water, the larvae have to maintain a body position that avoids friction against the stone surface, similar to the function of the adhesive disks of some species of *Rhithrogena* Eaton, 1881 and *Epeorus* Eaton, 1881 (Heptageniidae) (Ditsche-Kuru et al. 2010; Zhou 2010).

In conclusion, *Megabranchiella* gen. nov. from Thailand can be assigned to Acentrella/g1 Kluge & Novikova, 2011 based on the diagnostic morphological characters of the taxon. However, the complex taxonomy of Acentrella/g1 creates difficulties in deciding the position of this new genus within the clade. Molecular work can resolve and clarify the taxonomic status according to the evolutionary relationship with Acentrella/g1. The ecology and habit of this new genus make its association with Acentrella/g1 quite likely, in view of their similar habitats, behaviors, and morphology. A clearer picture of the adaptation and modifications of the gill morphological characteristics of this genus should be studied intensively in future work.

## Supplementary Material

XML Treatment for Megabranchiella

XML Treatment for Megabranchiella scutulata

XML Treatment for Megabranchiella longusa
